# Bis(2,2′-bipyridyl-κ^2^ *N*,*N*′)(sulfato-κ^2^ *O*,*O*′)nickel(II) 2.5-hydrate

**DOI:** 10.1107/S1600536813014219

**Published:** 2013-05-31

**Authors:** Turganbay Iskenderov

**Affiliations:** a Department of Chemistry, National Taras Shevchenko University, Volodymyrska Str. 64, 01601 Kiev, Ukraine

## Abstract

The title compound, [Ni(SO_4_)(C_10_H_8_N_2_)_2_]·2.5H_2_O, is a nickel(II) complex with a distorted octa­hedral coordination geometry. The Ni^II^ atom is bonded by two O atoms of the bidentate chelating sulfate ligand and the four N atoms of two chelating 2,2′-bi­pyridine ligands. The Ni—N bond lengths range from 2.059 (3) to 2.075 (3) Å and the Ni—O bond lengths are 2.098 (3) and 2.123 (3) Å. The bipyridyl ligands are both close to planar (r.m.s. deviations of 0.254 and 0.0572 Å) and are almost orthogonal, making a dihedral angle of 82.77 (1)°. In the crystal, the complex and water mol­ecules are connected by O—H⋯O hydrogen bonds. Inter­estingly, six water mol­ecules form a chain linking two complex mol­ecules *via* sulfate O atoms. There are also stacking inter­actions between the aromatic rings of neighbouring 2,2′-bi­pyridine ligands with shortest non-covalent contacts of 3.268 (6), 3.393 (6) and 3.435 (5) Å. One of the three unique water molecules shows half-occupation.

## Related literature

For applications of the 2,2′-bipyridyl ligand, see: Fritsky *et al.* (2004 ▶, 2006 ▶); Kanderal *et al.* (2005 ▶). For related structures, see: Fritsky *et al.* (1998 ▶, 2000 ▶); Moroz *et al.* (2010 ▶, 2012 ▶); Sliva *et al.* (1997 ▶); Świątek-Kozłowska *et al.* (2000 ▶); Iskenderov *et al.* (2009 ▶).
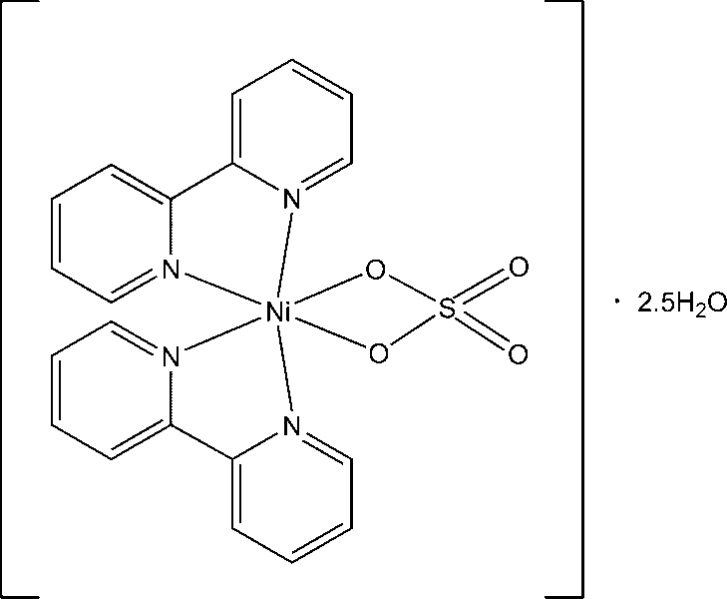


## Experimental

### 

#### Crystal data

[Ni(SO_4_)(C_10_H_8_N_2_)_2_]·2.5H_2_O
*M* *_r_* = 512.18
Triclinic, 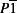

*a* = 10.045 (2) Å
*b* = 10.393 (2) Å
*c* = 11.028 (2) Å
α = 99.42 (3)°
β = 101.97 (3)°
γ = 98.56 (3)°
*V* = 1091.0 (4) Å^3^
*Z* = 2
Mo *K*α radiation
μ = 1.03 mm^−1^
*T* = 293 K
0.32 × 0.22 × 0.11 mm

#### Data collection

Enraf–Nonius CAD-4 diffractometer
Absorption correction: multi-scan (North *et al.*, 1968 ▶) *T*_min_ = 0.765, *T*_max_ = 0.897
3228 measured reflections
3021 independent reflections
2527 reflections with *I* > 2σ(*I*)
*R*_int_ = 0.030
3 standard reflections every 100 reflections intensity decay: 2%

#### Refinement

*R*[*F* ^2^ > 2σ(*F* ^2^)] = 0.042
*wR*(*F* ^2^) = 0.124
*S* = 1.07
3021 reflections
301 parameters
12 restraints
H-atom parameters constrained
Δρ_max_ = 0.76 e Å^−3^
Δρ_min_ = −0.44 e Å^−3^

### 

Data collection: *CAD-4 EXPRESS* (Enraf–Nonius, 1994 ▶); cell refinement: *CAD-4 EXPRESS*; data reduction: *XCAD4* (Harms & Wocadlo, 1995 ▶); program(s) used to solve structure: *SIR2004* (Burla *et al.*, 2005 ▶); program(s) used to refine structure: *SHELXL97* (Sheldrick, 2008 ▶); molecular graphics: *DIAMOND* (Brandenburg, 2009 ▶); software used to prepare material for publication: *SHELXL97*.

## Supplementary Material

Crystal structure: contains datablock(s) I, global. DOI: 10.1107/S1600536813014219/sj5321sup1.cif

Structure factors: contains datablock(s) I. DOI: 10.1107/S1600536813014219/sj5321Isup2.hkl

Additional supplementary materials:  crystallographic information; 3D view; checkCIF report

## Figures and Tables

**Table 1:** Hydrogen-bond geometry (Å, °)

*D*—H⋯*A*	*D*—H	H⋯*A*	*D*⋯*A*	*D*—H⋯*A*
O1*W*—H1*W*1⋯O1^i^	0.98	1.99	2.925 (5)	160
O1*W*—H2*W*1⋯O4^ii^	0.86	2.04	2.819 (5)	150
O2*W*—H1*W*2⋯O3*W* ^iii^	0.95	2.08	3.03 (2)	174
O2*W*—H2*W*2⋯O1*W*	0.85	1.93	2.774 (11)	170
O3*W*—H1*W*3⋯O3*W* ^iv^	0.99	1.91	2.86 (4)	163

Symmetry codes: (i) 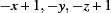; (ii) 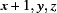; (iii) 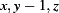; (iv) 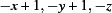.

**Fig. 1.**
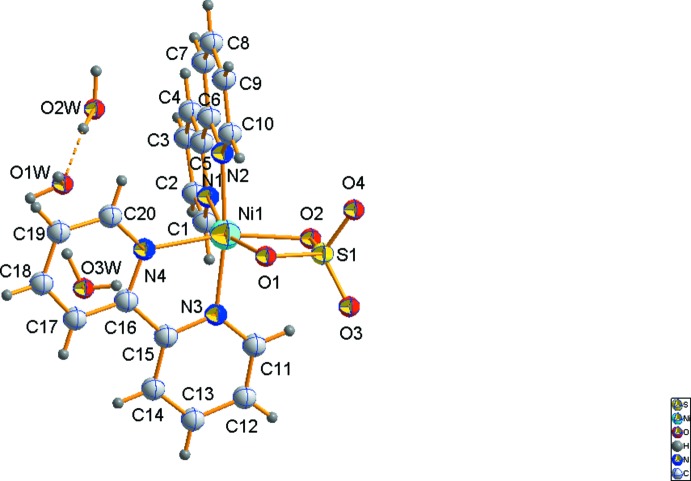
A view of the title compound, with displacement ellipsoids shown at the 50% probability level. H atoms are drawn as spheres of arbitrary radii.

**Fig. 2.**
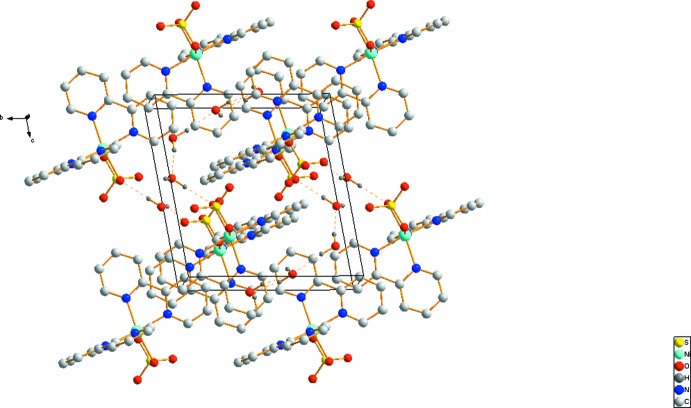
Crystal packing of the title compound. Hydrogen bonds are indicated by dashed lines. H atoms not involved in H-bonds are omitted for clarity.

## supplementary crystallographic information

### Comment

2,2'-bipyridyl (2,2'-bipy) is a well known neutral bidentate ligand which is widely used in coordination chemistry, in particular, for the preparation of mixed ligand complexes (Fritsky *et al.*, 2004; Kanderal *et al.*, 2005). It is also often used in the synthesis of discrete polynuclear complexes in order to prevent formation of coordination polymers by blocking a certain number of vacant sites in the coordination sphere of a metal ion (Fritsky *et al.*, 2006).

The asymmetric unit of title compound, comprises a neutral monomeric [Ni(SO_4_)(C_10_H_8_N_2_)_2_] complex and three solvent water molecules, one of which is present at half occupancy. The six-coordinate nickel(II) complex adopts a distorted octahedral coordination geometry (Fig. 1).The Ni^II^ atom is bonded by two O atoms of the bidentate chelating sulfate ligand and four N atoms of the two chelating 2,2'-bipyridine ligands. Each of the bipyridyl ligands is reasonably planar with rms deviations of 0.254Å and 0.0572Å from the best fit meanplanes through the non-hydrogen atoms of the N1,N2 and N3, N4 ligands respectively. The bipyridyl ligands are almost orthogonal [dihedral angle = 82.7 (1)°]. The Ni—N bond distances range from 2.059 (3) to 2.075 (3) Å and the Ni—O bond distances are 2.098 (3) and 2.123 (3) Å and are typical for distorted octahedral Ni^II^ complexes with the nitrogen and oxygen donors (Fritsky *et al.*, 1998; Świątek-Kozłowska *et al.*, 2000; Sliva *et al.*, 1997). The N—Ni—N bite angles around the central atom deviate significantly from 90°, [N2–Ni1–N1 = 78.94 (13)°, N3–Ni1–N4 = 79.32 (13)°], which is a consequence of the formation of five-membered chelate rings. The O2—Ni1—O1 bite angle is even smaller at 67.56 (10)° due to the formation of a four-membered chelate ring by the bidentate sulfate anion.

The C—N and C—C bond lengths in the pyridine rings are normal for 2-substituted pyridine derivatives (Fritsky *et al.*, 2000; Iskenderov *et al.*, 2009; Moroz *et al.*, 2010; Moroz *et al.*, 2012).

In the crystal structure, the [NiSO_4_(C_10_H_8_N_2_)_2_] and water molecules are connected by intermolecular O—H···O hydrogen bonding, (Fig. 2) in which the water molecules act as donors while the sulfate anions and water oxygen atoms act as acceptors (Fig. 2). Interestingly, six water molecules form a chain O1W···O2W···O3W···O3W'···O2W'···O1W' linking two complex molecules via the O(4) atoms of the sulfate anions. There are also stacking interactions between the aromatic rings of the 2,2'-bipyridine molecules belonging to the neighboring complex molecules with shortest non-covalent contacts C(12)···C(18) (1-x, 1-y, 1-z) = 3.268 (6)Å; C(11)···C(18) = 3.393 (6)Å and N(3)···C(17) (1-x, 1-y, 1-z) = 3.435 (5) Å (Fig. 2).

### Experimental

Nickel(II) sulfate hexahydrate (0.026 g, 0.1 mmol) was dissolved in methanol (5 ml) and mixed with a solution of 2,2'-bipyridine (0.312 g, 2 mmol) in methanol (5 ml), afterwards the resulting transparent blue solution was left to evaporate in the air at ambient temperature. During 12 h the blue polycrystalline product precipitated from the solution. It was filtered off, washed with diethyl ether and dried in the air. Yield: 85%. Elemental analysis calc. (%) for C_20_H_22_N_4_NiO_7_S: C 46.09; H 4.25; N 10.75; found: C 47.11; H 4.60; N 10.43.

### Refinement

As the data were collected on an older diffractometer, collection ceased once sufficient reflections had been obtained to adequately solve and refine the structure, hence the number of missing reflections indicated in the B alert. The H_2_O H atoms of the solvate water molecules were located from the difference Fourier map but constrained to ride on their parent atom, with *U*_iso_ = 1.5 *U*_eq_(parent atom). One of the solvate water molecules was refined with an occupancy factor of 0.5 (an attempt to refine the occupancy factor freely converged with a value of 0.5). The C—H atoms were positioned geometrically and refined as riding atoms, with C—H = 0.93 and *U*_iso_ = 1.2*U*_eq_(parent atom).

### Crystal data

**Table:**

[Ni(SO_4_)(C_10_H_8_N_2_)_2_]·2.5H_2_O	*Z* = 2
*M**_r_* = 512.18	*F*(000) = 530
Triclinic, *P*1	*D*_x_ = 1.559 Mg m^−^^3^
Hall symbol: -P 1	Mo *K*α radiation, λ = 0.71073 Å
*a* = 10.045 (2) Å	Cell parameters from 1565 reflections
*b* = 10.393 (2) Å	θ = 3.0–25.5°
*c* = 11.028 (2) Å	µ = 1.03 mm^−^^1^
α = 99.42 (3)°	*T* = 293 K
β = 101.97 (3)°	Block, blue
γ = 98.56 (3)°	0.32 × 0.22 × 0.11 mm
*V* = 1091.0 (4) Å^3^	

### Data collection

**Table:**

Enraf–Nonius CAD-4 diffractometer	3021 independent reflections
Radiation source: fine-focus sealed tube	2527 reflections with *I* > 2σ(*I*)
Horizontally mounted graphite crystal monochromator	*R*_int_ = 0.030
Detector resolution: 9 pixels mm^-1^	θ_max_ = 26.1°, θ_min_ = 1.9°
profile data from ω/2θ scans	*h* = 0→12
Absorption correction: ψ scan (North *et al.*, 1968)	*k* = −12→12
*T*_min_ = 0.765, *T*_max_ = 0.897	*l* = −12→12
3228 measured reflections	

### Refinement

**Table:**

Refinement on *F*^2^	Primary atom site location: structure-invariant direct methods
Least-squares matrix: full	Secondary atom site location: difference Fourier map
*R*[*F*^2^ > 2σ(*F*^2^)] = 0.042	Hydrogen site location: inferred from neighbouring sites
*wR*(*F*^2^) = 0.124	H-atom parameters constrained
*S* = 1.07	*w* = 1/[σ^2^(*F*_o_^2^) + (0.0784*P*)^2^ + 0.7731*P*] where *P* = (*F*_o_^2^ + 2*F*_c_^2^)/3
3021 reflections	(Δ/σ)_max_ < 0.001
301 parameters	Δρ_max_ = 0.76 e Å^−^^3^
12 restraints	Δρ_min_ = −0.44 e Å^−^^3^

### Special details

**Table:**

Experimental. The H_2_O H atoms of the solvate water molecules were located from the difference Fourier map but constrained to ride on their parent atom, with *U*_iso_ = 1.5 *U*_eq_(parent atom). One of the solvate water molecule was included into refinement with the occupancy factor of 0.5 (as an attempt to refine it with free variation of the occupancy factor resulted in its value of 1/2, and as O3W forms an H-bond with the tranlsational O3W water molecule through the hydrogen atom H1W3 which limits the occupancy of the latter by 1/2). The C—H atoms were positioned geometrically and refined as riding atoms, with C—H = 0.93 and *U*_iso_ = 1.2*U*_eq_(parent atom).
Geometry. All e.s.d.'s (except the e.s.d. in the dihedral angle between two l.s. planes) are estimated using the full covariance matrix. The cell e.s.d.'s are taken into account individually in the estimation of e.s.d.'s in distances, angles and torsion angles; correlations between e.s.d.'s in cell parameters are only used when they are defined by crystal symmetry. An approximate (isotropic) treatment of cell e.s.d.'s is used for estimating e.s.d.'s involving l.s. planes.
Refinement. Refinement of *F*^2^ against ALL reflections. The weighted *R*-factor *wR* and goodness of fit *S* are based on *F*^2^, conventional *R*-factors *R* are based on *F*, with *F* set to zero for negative *F*^2^. The threshold expression of *F*^2^ > σ(*F*^2^) is used only for calculating *R*-factors(gt) *etc*. and is not relevant to the choice of reflections for refinement. *R*-factors based on *F*^2^ are statistically about twice as large as those based on *F*, and *R*- factors based on ALL data will be even larger.

### Fractional atomic coordinates and isotropic or equivalent isotropic displacement parameters (Å^2^)

**Table:**

	*x*	*y*	*z*	*U*_iso_*/*U*_eq_	Occ. (<1)
S1	0.05920 (10)	0.23826 (10)	0.38907 (9)	0.0340 (3)	
Ni1	0.23116 (5)	0.29554 (5)	0.24003 (4)	0.0288 (2)	
O1	0.2111 (3)	0.2317 (3)	0.4097 (2)	0.0345 (6)	
O2	0.0295 (3)	0.2855 (3)	0.2672 (2)	0.0342 (6)	
O3	0.0352 (3)	0.3311 (3)	0.4914 (3)	0.0478 (8)	
O4	−0.0214 (3)	0.1049 (3)	0.3732 (3)	0.0548 (9)	
O1W	0.7471 (4)	−0.0268 (4)	0.4408 (4)	0.0751 (11)	
H1W1	0.7839	−0.0894	0.4895	0.113*	
H2W1	0.8304	0.0141	0.4490	0.113*	
O2W	0.5607 (10)	−0.0896 (10)	0.2062 (10)	0.225 (4)	
H1W2	0.5395	−0.1799	0.1620	0.337*	
H2W2	0.6204	−0.0800	0.2757	0.337*	
O3W	0.482 (2)	0.631 (2)	0.0491 (18)	0.237 (9)	0.50
H1W3	0.5088	0.5500	0.0081	0.356*	0.50
H2W3	0.4120	0.6030	0.0716	0.356*	0.50
N1	0.1856 (3)	0.3201 (3)	0.0539 (3)	0.0354 (8)	
N2	0.2016 (3)	0.1024 (3)	0.1437 (3)	0.0324 (7)	
N3	0.2837 (3)	0.4950 (3)	0.3228 (3)	0.0300 (7)	
N4	0.4444 (3)	0.3267 (3)	0.2655 (3)	0.0312 (7)	
C1	0.1708 (5)	0.4330 (4)	0.0137 (4)	0.0459 (11)	
H1	0.1829	0.5105	0.0737	0.055*	
C2	0.1387 (6)	0.4396 (5)	−0.1111 (4)	0.0542 (13)	
H2	0.1279	0.5198	−0.1353	0.065*	
C3	0.1227 (5)	0.3263 (5)	−0.2006 (4)	0.0475 (11)	
H3	0.1034	0.3288	−0.2862	0.057*	
C4	0.1356 (5)	0.2089 (4)	−0.1611 (4)	0.0410 (10)	
H4	0.1237	0.1306	−0.2201	0.049*	
C5	0.1663 (4)	0.2082 (4)	−0.0339 (4)	0.0324 (9)	
C6	0.1766 (4)	0.0860 (4)	0.0172 (4)	0.0313 (9)	
C7	0.1606 (5)	−0.0371 (4)	−0.0592 (4)	0.0418 (10)	
H7	0.1424	−0.0468	−0.1467	0.050*	
C8	0.1722 (5)	−0.1456 (4)	−0.0033 (4)	0.0495 (12)	
H8	0.1614	−0.2296	−0.0530	0.059*	
C9	0.1998 (5)	−0.1289 (4)	0.1260 (4)	0.0465 (11)	
H9	0.2099	−0.2007	0.1653	0.056*	
C10	0.2123 (5)	−0.0038 (4)	0.1963 (4)	0.0416 (10)	
H10	0.2288	0.0074	0.2838	0.050*	
C11	0.1965 (4)	0.5742 (4)	0.3500 (4)	0.0359 (9)	
H11	0.1029	0.5374	0.3345	0.043*	
C12	0.2385 (4)	0.7071 (4)	0.3996 (4)	0.0401 (10)	
H12	0.1745	0.7595	0.4164	0.048*	
C13	0.3771 (5)	0.7620 (4)	0.4242 (4)	0.0471 (11)	
H13	0.4081	0.8525	0.4566	0.057*	
C14	0.4694 (5)	0.6806 (4)	0.3999 (4)	0.0447 (11)	
H14	0.5638	0.7152	0.4174	0.054*	
C15	0.4197 (4)	0.5473 (4)	0.3494 (3)	0.0327 (9)	
C16	0.5110 (4)	0.4517 (4)	0.3221 (3)	0.0327 (9)	
C17	0.6536 (4)	0.4860 (4)	0.3560 (4)	0.0398 (10)	
H17	0.6972	0.5725	0.3954	0.048*	
C18	0.7305 (5)	0.3903 (5)	0.3306 (4)	0.0470 (11)	
H18	0.8268	0.4114	0.3538	0.056*	
C19	0.6657 (5)	0.2657 (5)	0.2719 (4)	0.0494 (12)	
H19	0.7163	0.2004	0.2526	0.059*	
C20	0.5228 (4)	0.2372 (4)	0.2410 (4)	0.0421 (10)	
H20	0.4787	0.1510	0.2010	0.051*	

### Atomic displacement parameters (Å^2^)

**Table:**

	*U*^11^	*U*^22^	*U*^33^	*U*^12^	*U*^13^	*U*^23^
S1	0.0296 (6)	0.0366 (6)	0.0294 (5)	−0.0008 (4)	0.0026 (4)	0.0003 (4)
Ni1	0.0278 (3)	0.0261 (3)	0.0256 (3)	−0.0002 (2)	0.0010 (2)	−0.0033 (2)
O1	0.0318 (15)	0.0384 (15)	0.0293 (14)	0.0058 (12)	0.0018 (12)	0.0038 (12)
O2	0.0278 (14)	0.0357 (15)	0.0313 (15)	0.0009 (12)	−0.0034 (12)	0.0026 (12)
O3	0.0407 (17)	0.063 (2)	0.0349 (16)	0.0123 (15)	0.0082 (13)	−0.0041 (14)
O4	0.053 (2)	0.0452 (18)	0.056 (2)	−0.0144 (15)	0.0058 (16)	0.0120 (16)
O1W	0.065 (2)	0.064 (2)	0.103 (3)	0.0146 (19)	0.017 (2)	0.036 (2)
O2W	0.204 (7)	0.213 (7)	0.244 (8)	0.038 (6)	0.016 (6)	0.058 (6)
O3W	0.237 (12)	0.248 (12)	0.222 (12)	0.012 (9)	0.059 (9)	0.057 (9)
N1	0.0380 (19)	0.0312 (18)	0.0312 (18)	0.0005 (15)	0.0042 (15)	0.0007 (15)
N2	0.0331 (18)	0.0304 (17)	0.0281 (18)	0.0024 (14)	0.0015 (14)	0.0006 (14)
N3	0.0271 (18)	0.0314 (17)	0.0264 (17)	0.0023 (14)	0.0018 (14)	0.0000 (13)
N4	0.0309 (17)	0.0341 (18)	0.0252 (16)	0.0058 (15)	0.0036 (14)	0.0012 (14)
C1	0.061 (3)	0.036 (2)	0.037 (2)	0.006 (2)	0.005 (2)	0.0060 (19)
C2	0.078 (4)	0.039 (3)	0.045 (3)	0.011 (2)	0.011 (3)	0.012 (2)
C3	0.054 (3)	0.056 (3)	0.031 (2)	0.007 (2)	0.007 (2)	0.012 (2)
C4	0.047 (3)	0.040 (2)	0.029 (2)	0.004 (2)	0.0042 (19)	−0.0028 (18)
C5	0.030 (2)	0.033 (2)	0.029 (2)	0.0028 (17)	0.0041 (17)	−0.0027 (17)
C6	0.026 (2)	0.033 (2)	0.030 (2)	0.0015 (17)	0.0029 (16)	−0.0012 (17)
C7	0.047 (3)	0.039 (2)	0.031 (2)	0.006 (2)	0.0030 (19)	−0.0060 (18)
C8	0.060 (3)	0.029 (2)	0.049 (3)	0.002 (2)	0.005 (2)	−0.005 (2)
C9	0.057 (3)	0.031 (2)	0.047 (3)	0.005 (2)	0.006 (2)	0.003 (2)
C10	0.046 (3)	0.039 (2)	0.035 (2)	0.004 (2)	0.0043 (19)	0.0034 (19)
C11	0.031 (2)	0.036 (2)	0.037 (2)	0.0028 (18)	0.0057 (18)	0.0021 (18)
C12	0.045 (3)	0.032 (2)	0.041 (2)	0.012 (2)	0.009 (2)	0.0012 (18)
C13	0.054 (3)	0.031 (2)	0.046 (3)	0.000 (2)	0.003 (2)	−0.0045 (19)
C14	0.037 (2)	0.036 (2)	0.046 (3)	−0.009 (2)	−0.001 (2)	−0.004 (2)
C15	0.034 (2)	0.035 (2)	0.025 (2)	0.0027 (18)	0.0016 (17)	0.0051 (17)
C16	0.030 (2)	0.040 (2)	0.0235 (19)	−0.0003 (18)	0.0037 (16)	0.0041 (17)
C17	0.028 (2)	0.048 (3)	0.038 (2)	0.0006 (19)	0.0021 (18)	0.007 (2)
C18	0.029 (2)	0.065 (3)	0.047 (3)	0.009 (2)	0.007 (2)	0.015 (2)
C19	0.041 (3)	0.063 (3)	0.047 (3)	0.021 (2)	0.011 (2)	0.005 (2)
C20	0.038 (2)	0.038 (2)	0.046 (3)	0.008 (2)	0.008 (2)	−0.004 (2)

### Geometric parameters (Å, º)

**Table:**

S1—O3	1.445 (3)	C3—H3	0.9300
S1—O4	1.461 (3)	C4—C5	1.374 (6)
S1—O2	1.493 (3)	C4—H4	0.9300
S1—O1	1.509 (3)	C5—C6	1.480 (6)
S1—Ni1	2.6892 (13)	C6—C7	1.380 (6)
Ni1—N2	2.059 (3)	C7—C8	1.379 (6)
Ni1—N3	2.065 (3)	C7—H7	0.9300
Ni1—N4	2.070 (3)	C8—C9	1.372 (6)
Ni1—N1	2.075 (3)	C8—H8	0.9300
Ni1—O2	2.098 (3)	C9—C10	1.374 (6)
Ni1—O1	2.123 (3)	C9—H9	0.9300
O1W—H1W1	0.9753	C10—H10	0.9300
O1W—H2W1	0.8603	C11—C12	1.367 (6)
O2W—H1W2	0.9549	C11—H11	0.9300
O2W—H2W2	0.8500	C12—C13	1.376 (6)
O3W—H1W3	0.9888	C12—H12	0.9300
O3W—H2W3	0.8245	C13—C14	1.382 (6)
N1—C1	1.338 (5)	C13—H13	0.9300
N1—C5	1.347 (5)	C14—C15	1.380 (6)
N2—C10	1.335 (5)	C14—H14	0.9300
N2—C6	1.342 (5)	C15—C16	1.485 (6)
N3—C11	1.334 (5)	C16—C17	1.379 (6)
N3—C15	1.345 (5)	C17—C18	1.375 (6)
N4—C20	1.335 (5)	C17—H17	0.9300
N4—C16	1.352 (5)	C18—C19	1.349 (7)
C1—C2	1.362 (6)	C18—H18	0.9300
C1—H1	0.9300	C19—C20	1.379 (6)
C2—C3	1.372 (7)	C19—H19	0.9300
C2—H2	0.9300	C20—H20	0.9300
C3—C4	1.376 (6)		
			
O3—S1—O4	112.5 (2)	C2—C3—C4	118.7 (4)
O3—S1—O2	111.38 (18)	C2—C3—H3	120.6
O4—S1—O2	109.64 (17)	C4—C3—H3	120.6
O3—S1—O1	110.62 (17)	C5—C4—C3	119.5 (4)
O4—S1—O1	109.38 (18)	C5—C4—H4	120.3
O2—S1—O1	102.89 (16)	C3—C4—H4	120.3
O3—S1—Ni1	125.58 (14)	N1—C5—C4	121.7 (4)
O4—S1—Ni1	121.92 (15)	N1—C5—C6	115.0 (3)
O2—S1—Ni1	50.95 (11)	C4—C5—C6	123.3 (4)
O1—S1—Ni1	51.94 (11)	N2—C6—C7	121.8 (4)
N2—Ni1—N3	171.54 (12)	N2—C6—C5	115.5 (3)
N2—Ni1—N4	93.94 (13)	C7—C6—C5	122.7 (4)
N3—Ni1—N4	79.32 (13)	C8—C7—C6	118.7 (4)
N2—Ni1—N1	78.92 (13)	C8—C7—H7	120.6
N3—Ni1—N1	96.65 (13)	C6—C7—H7	120.6
N4—Ni1—N1	96.66 (13)	C9—C8—C7	119.6 (4)
N2—Ni1—O2	96.16 (12)	C9—C8—H8	120.2
N3—Ni1—O2	91.51 (12)	C7—C8—H8	120.2
N4—Ni1—O2	164.38 (11)	C8—C9—C10	118.6 (4)
N1—Ni1—O2	96.95 (12)	C8—C9—H9	120.7
N2—Ni1—O1	91.50 (12)	C10—C9—H9	120.7
N3—Ni1—O1	94.73 (12)	N2—C10—C9	122.6 (4)
N4—Ni1—O1	100.34 (12)	N2—C10—H10	118.7
N1—Ni1—O1	161.03 (12)	C9—C10—H10	118.7
O2—Ni1—O1	67.57 (10)	N3—C11—C12	123.0 (4)
N2—Ni1—S1	94.37 (10)	N3—C11—H11	118.5
N3—Ni1—S1	93.99 (9)	C12—C11—H11	118.5
N4—Ni1—S1	133.72 (9)	C11—C12—C13	118.9 (4)
N1—Ni1—S1	129.62 (10)	C11—C12—H12	120.6
O2—Ni1—S1	33.54 (7)	C13—C12—H12	120.6
O1—Ni1—S1	34.03 (7)	C12—C13—C14	118.9 (4)
S1—O1—Ni1	94.03 (13)	C12—C13—H13	120.5
S1—O2—Ni1	95.51 (13)	C14—C13—H13	120.5
H1W1—O1W—H2W1	89.2	C15—C14—C13	119.1 (4)
H1W2—O2W—H2W2	110.8	C15—C14—H14	120.4
H1W3—O3W—H2W3	104.2	C13—C14—H14	120.4
C1—N1—C5	118.0 (4)	N3—C15—C14	121.6 (4)
C1—N1—Ni1	127.0 (3)	N3—C15—C16	115.4 (3)
C5—N1—Ni1	115.0 (3)	C14—C15—C16	123.0 (4)
C10—N2—C6	118.7 (3)	N4—C16—C17	122.0 (4)
C10—N2—Ni1	125.8 (3)	N4—C16—C15	115.2 (3)
C6—N2—Ni1	115.4 (3)	C17—C16—C15	122.7 (4)
C11—N3—C15	118.5 (3)	C18—C17—C16	119.0 (4)
C11—N3—Ni1	126.4 (3)	C18—C17—H17	120.5
C15—N3—Ni1	115.1 (3)	C16—C17—H17	120.5
C20—N4—C16	117.1 (3)	C19—C18—C17	119.7 (4)
C20—N4—Ni1	127.9 (3)	C19—C18—H18	120.1
C16—N4—Ni1	114.8 (3)	C17—C18—H18	120.1
N1—C1—C2	123.0 (4)	C18—C19—C20	118.6 (4)
N1—C1—H1	118.5	C18—C19—H19	120.7
C2—C1—H1	118.5	C20—C19—H19	120.7
C1—C2—C3	119.1 (4)	N4—C20—C19	123.5 (4)
C1—C2—H2	120.5	N4—C20—H20	118.2
C3—C2—H2	120.5	C19—C20—H20	118.2
			
O3—S1—Ni1—N2	175.62 (18)	N4—Ni1—N3—C15	1.3 (3)
O4—S1—Ni1—N2	−4.30 (19)	N1—Ni1—N3—C15	−94.2 (3)
O2—S1—Ni1—N2	−94.56 (16)	O2—Ni1—N3—C15	168.6 (3)
O1—S1—Ni1—N2	86.20 (16)	O1—Ni1—N3—C15	101.0 (3)
O3—S1—Ni1—N3	−3.11 (18)	S1—Ni1—N3—C15	135.1 (3)
O4—S1—Ni1—N3	176.96 (18)	N2—Ni1—N4—C20	−8.9 (4)
O2—S1—Ni1—N3	86.71 (16)	N3—Ni1—N4—C20	176.3 (4)
O1—S1—Ni1—N3	−92.54 (16)	N1—Ni1—N4—C20	−88.2 (3)
O3—S1—Ni1—N4	75.9 (2)	O2—Ni1—N4—C20	121.3 (5)
O4—S1—Ni1—N4	−104.0 (2)	O1—Ni1—N4—C20	83.3 (3)
O2—S1—Ni1—N4	165.74 (18)	S1—Ni1—N4—C20	91.0 (3)
O1—S1—Ni1—N4	−13.50 (18)	N2—Ni1—N4—C16	176.2 (3)
O3—S1—Ni1—N1	−105.1 (2)	N3—Ni1—N4—C16	1.3 (3)
O4—S1—Ni1—N1	74.9 (2)	N1—Ni1—N4—C16	96.9 (3)
O2—S1—Ni1—N1	−15.33 (18)	O2—Ni1—N4—C16	−53.6 (5)
O1—S1—Ni1—N1	165.43 (17)	O1—Ni1—N4—C16	−91.6 (3)
O3—S1—Ni1—O2	−89.8 (2)	S1—Ni1—N4—C16	−83.9 (3)
O4—S1—Ni1—O2	90.3 (2)	C5—N1—C1—C2	−0.7 (7)
O1—S1—Ni1—O2	−179.24 (19)	Ni1—N1—C1—C2	−179.1 (4)
O3—S1—Ni1—O1	89.4 (2)	N1—C1—C2—C3	−1.0 (8)
O4—S1—Ni1—O1	−90.5 (2)	C1—C2—C3—C4	1.8 (7)
O2—S1—Ni1—O1	179.24 (19)	C2—C3—C4—C5	−1.0 (7)
O3—S1—O1—Ni1	−119.66 (17)	C1—N1—C5—C4	1.6 (6)
O4—S1—O1—Ni1	115.88 (17)	Ni1—N1—C5—C4	−179.9 (3)
O2—S1—O1—Ni1	−0.60 (15)	C1—N1—C5—C6	−176.7 (4)
N2—Ni1—O1—S1	−95.60 (15)	Ni1—N1—C5—C6	1.8 (4)
N3—Ni1—O1—S1	90.14 (14)	C3—C4—C5—N1	−0.7 (6)
N4—Ni1—O1—S1	170.12 (13)	C3—C4—C5—C6	177.4 (4)
N1—Ni1—O1—S1	−36.6 (4)	C10—N2—C6—C7	0.6 (6)
O2—Ni1—O1—S1	0.45 (11)	Ni1—N2—C6—C7	177.2 (3)
O3—S1—O2—Ni1	119.14 (16)	C10—N2—C6—C5	−179.9 (3)
O4—S1—O2—Ni1	−115.69 (18)	Ni1—N2—C6—C5	−3.3 (4)
O1—S1—O2—Ni1	0.61 (15)	N1—C5—C6—N2	1.0 (5)
N2—Ni1—O2—S1	88.62 (15)	C4—C5—C6—N2	−177.3 (4)
N3—Ni1—O2—S1	−94.95 (14)	N1—C5—C6—C7	−179.5 (4)
N4—Ni1—O2—S1	−41.4 (5)	C4—C5—C6—C7	2.2 (6)
N1—Ni1—O2—S1	168.16 (14)	N2—C6—C7—C8	−0.7 (6)
O1—Ni1—O2—S1	−0.46 (11)	C5—C6—C7—C8	179.9 (4)
N2—Ni1—N1—C1	175.7 (4)	C6—C7—C8—C9	−0.3 (7)
N3—Ni1—N1—C1	−11.6 (4)	C7—C8—C9—C10	1.3 (7)
N4—Ni1—N1—C1	−91.6 (4)	C6—N2—C10—C9	0.5 (6)
O2—Ni1—N1—C1	80.7 (4)	Ni1—N2—C10—C9	−175.7 (3)
O1—Ni1—N1—C1	114.8 (4)	C8—C9—C10—N2	−1.5 (7)
S1—Ni1—N1—C1	89.2 (4)	C15—N3—C11—C12	2.3 (6)
N2—Ni1—N1—C5	−2.7 (3)	Ni1—N3—C11—C12	−176.9 (3)
N3—Ni1—N1—C5	170.0 (3)	N3—C11—C12—C13	−0.8 (6)
N4—Ni1—N1—C5	90.0 (3)	C11—C12—C13—C14	−1.1 (7)
O2—Ni1—N1—C5	−97.7 (3)	C12—C13—C14—C15	1.3 (7)
O1—Ni1—N1—C5	−63.5 (5)	C11—N3—C15—C14	−2.0 (6)
S1—Ni1—N1—C5	−89.2 (3)	Ni1—N3—C15—C14	177.2 (3)
N3—Ni1—N2—C10	120.5 (8)	C11—N3—C15—C16	177.2 (3)
N4—Ni1—N2—C10	83.6 (3)	Ni1—N3—C15—C16	−3.5 (4)
N1—Ni1—N2—C10	179.6 (4)	C13—C14—C15—N3	0.2 (6)
O2—Ni1—N2—C10	−84.5 (3)	C13—C14—C15—C16	−178.9 (4)
O1—Ni1—N2—C10	−16.9 (3)	C20—N4—C16—C17	−1.3 (6)
S1—Ni1—N2—C10	−50.8 (3)	Ni1—N4—C16—C17	174.2 (3)
N3—Ni1—N2—C6	−55.8 (9)	C20—N4—C16—C15	−179.0 (3)
N4—Ni1—N2—C6	−92.8 (3)	Ni1—N4—C16—C15	−3.5 (4)
N1—Ni1—N2—C6	3.3 (3)	N3—C15—C16—N4	4.7 (5)
O2—Ni1—N2—C6	99.2 (3)	C14—C15—C16—N4	−176.1 (4)
O1—Ni1—N2—C6	166.8 (3)	N3—C15—C16—C17	−173.0 (4)
S1—Ni1—N2—C6	132.8 (3)	C14—C15—C16—C17	6.2 (6)
N2—Ni1—N3—C11	142.9 (7)	N4—C16—C17—C18	0.5 (6)
N4—Ni1—N3—C11	−179.5 (3)	C15—C16—C17—C18	178.0 (4)
N1—Ni1—N3—C11	84.9 (3)	C16—C17—C18—C19	0.9 (7)
O2—Ni1—N3—C11	−12.2 (3)	C17—C18—C19—C20	−1.3 (7)
O1—Ni1—N3—C11	−79.8 (3)	C16—N4—C20—C19	0.9 (6)
S1—Ni1—N3—C11	−45.7 (3)	Ni1—N4—C20—C19	−173.9 (3)
N2—Ni1—N3—C15	−36.3 (9)	C18—C19—C20—N4	0.4 (7)

### Hydrogen-bond geometry (Å, º)

**Table:**

*D*—H···*A*	*D*—H	H···*A*	*D*···*A*	*D*—H···*A*
O1*W*—H1*W*1···O1^i^	0.98	1.99	2.925 (5)	160
O1*W*—H2*W*1···O4^ii^	0.86	2.04	2.819 (5)	150
O2*W*—H1*W*2···O3*W*^iii^	0.95	2.08	3.03 (2)	174
O2*W*—H2*W*2···O1*W*	0.85	1.93	2.774 (11)	170
O3*W*—H1*W*3···O3*W*^iv^	0.99	1.91	2.86 (4)	163

Symmetry codes: (i) −*x*+1, −*y*, −*z*+1; (ii) *x*+1, *y*, *z*; (iii) *x*, *y*−1, *z*; (iv) −*x*+1, −*y*+1, −*z*.
